# Early Prognostication After Out-of-Hospital Cardiac Arrest: Modified rCAST Score Incorporating Age and Brainstem Reflexes

**DOI:** 10.3390/jcm14196830

**Published:** 2025-09-26

**Authors:** Youn-Jung Kim, Yonghun Jung, Byung Kook Lee, Chun Song Youn, Won Young Kim

**Affiliations:** 1 Department of Emergency Medicine, Asan Medical Center, Ulsan University College of Medicine, Seoul 05505, Republic of Korea; yjkim.em@amc.seoul.kr; 2 Department of Emergency Medicine, Chonnam National University Hospital, Chonnam National University Medical School, Gwangju 61469, Republic of Korea; xnxn77@hanmail.net (Y.J.); bbukkuk@hanmail.net (B.K.L.); 3 Departments of Emergency Medicine, Catholic University of Korea, Seoul 06591, Republic of Korea; ycs1005@catholic.ac.kr

**Keywords:** cardiac arrest, post-cardiac arrest syndrome, risk stratification, neurological outcome, prognosis

## Abstract

**Background**: Out-of-hospital cardiac arrest (OHCA) survivors demonstrate wide variation in neurological outcomes due to hypoxic–ischemic brain injury. Early prognostic stratification in the emergency department is essential to inform clinical decisions. This study aimed to improve the revised Cardiac Arrest Syndrome for Therapeutic hypothermia (rCAST) score by incorporating additional clinical variables and to evaluate its ability to predict poor neurological outcomes. **Methods**: This multicenter observational study analyzed OHCA survivors treated with targeted temperature management (TTM) between October 2015 and December 2018 at 22 university-affiliated hospitals participating in the Korean Hypothermia Network prospective registry. The primary outcome was poor neurological status at one month, defined as a Cerebral Performance Category (CPC) score of 3–5. Independent predictors were identified using multivariable logistic regression and incorporated into a modified rCAST (mCAST) score. **Results**: Among 881 included patients, age > 65 years (odds ratio [OR], 13.87; 95% confidence interval [CI], 7.38–26.08) and absence of brainstem reflexes (OR, 2.31; 95% CI, 1.29–4.12) were identified as independent predictors and added to the mCAST score. The mCAST demonstrated higher prognostic accuracy than the original rCAST (area under the curve [AUC], 0.849 vs. 0.823; *p* < 0.001). In the high-severity group, the mCAST identified a higher poor outcome rate (95.1% vs. 90.9%) while reducing the proportion of patients in this group (20.7% vs. 31.3%). **Conclusions**: The mCAST score improves early prognostic accuracy during the immediate post-cardiac arrest period by incorporating age and brainstem reflexes and may offer refined risk stratification without compromising clinical feasibility.

## 1. Introduction

Return of spontaneous circulation (ROSC) following out-of-hospital cardiac arrest (OHCA) encompasses a broad spectrum of prognostic trajectories, reflecting the variable extent of hypoxic–ischemic brain injury sustained during the arrest. This variability is predominantly influenced by factors such as the duration of circulatory arrest and its underlying etiology. As a result, the post-ROSC population includes patients with irreversible brain injury, those with minimal injury who are likely to recover independently of intensive interventions, and an intermediate subset in whom targeted treatment strategies may alter outcomes. Given this heterogeneity, accurate and timely risk stratification during the early post-resuscitation phase, particularly in the emergency department before intensive care unit admission, is essential. Such early assessment can guide clinical decisions, improve communications with families, optimize the allocation of critical care resources, and mitigate the risks associated with both under- and over-treatment.

The revised post–Cardiac Arrest Syndrome for Therapeutic hypothermia (rCAST) score was developed to facilitate such stratification using five parameters that are readily obtainable during the immediate post-cardiac arrest period: initial rhythm, witnessed status and time to ROSC, arterial pH, serum lactate concentration, and Glasgow Coma Scale (GCS) motor score [1,2,3]. Although previous studies have demonstrated the predictive utility of the rCAST score in estimating neurological outcomes among comatose OHCA survivors, its performance may still be improved [3,4,5,6,7]. Even incremental enhancements in prognostic precision hold significant clinical relevance, particularly in preventing premature withdrawal of life-sustaining therapy (WLST) or the inappropriate continuation of futile care. Consequently, efforts to refine existing prognostic models are warranted to achieve a balance between predictive accuracy and practical feasibility in the intensive care setting.

In this present study, we aimed to improve the prognostic capacity of the rCAST score by incorporating additional variables that are readily available during the immediate post-cardiac arrest period. We thereby developed a modified version of the rCAST (mCAST) score and evaluated its discriminatory performance for predicting poor neurological outcomes at one month in a prospectively enrolled multicenter cohort of comatose OHCA survivors treated with targeted temperature management (TTM).

## 2. Materials and Methods

### 2.1. Study Design and Population

This study was a retrospective analysis based on data obtained from the Korean Hypothermia Network prospective registry (KORHN-PRO), a nationwide, multicenter, observational cohort enrolling comatose adult patients resuscitated from OHCA [8]. Between October 2015 and December 2018, a total of 22 university-affiliated teaching hospitals across South Korea contributed data to the registry. The KORHN-PRO registry collected 136 variables with 839 datasets and was registered at ClinicalTrials.gov (NCT02827422). Patients eligible for inclusion were adults (aged > 18 years) who remained comatose after OHCA and were treated with TTM. Patients were excluded if they had a terminal illness with an expected survival of less than six months (as documented in medical records), were under hospice care, had experienced an intracranial hemorrhage or acute stroke, possessed a pre-existing “Do Not Resuscitate” order, had a cerebral performance category (CPC) score of 3 or 4 prior to the cardiac arrest, or presented with a core body temperature below 30 °C on admission. The institutional review board of each of the participating hospitals reviewed and approved the study protocol, including that of Asan Medical Center (No. 2019-1204) and the investigators obtained written informed consent from legal surrogates prior to subject enrollment due to the patient’s comatose state.

For the present analysis, we included patients within the registry who experienced a cardiac arrest of presumed medical etiology, for whom calculation of the rCAST score was feasible, and whose neurological outcome at one month post-arrest was available and assessed using the CPC scale.

### 2.2. Patient Management

All enrolled patients received standardized post-resuscitation care in accordance with the then-current international guidelines [9,10]. TTM was initiated using either surface or endovascular cooling devices, including Blanketrol II (Cincinnati Subzero Products, Cincinnati, OH, USA), Arctic Sun Energy Transfer Pad (Medivance Corp., Louisville, CO, USA), or Thermoguard (ZOLL Medical Corporation, Chelmsford, MA, USA). Core body temperature was maintained between 32 and 36 °C for 24 h, with the target temperature selected at the discretion of the treating intensivist. Rewarming was performed at a controlled rate of 0.25–0.5 °C/h. Normothermia (37 °C) was maintained for 72 h after ROSC. The decision to administer sedatives and analgesic agents such as propofol, remifentanil, morphine, midazolam, or fentanyl was made by the respective intensivist in accordance with each institution’s clinical protocol. Neuromuscular blocking agents were administered as needed to control shivering or ventilator–patient dyssynchrony.

Active WLST had been legally prohibited in South Korea until February 2018, and the enrolled patients in this present study had therefore received full supportive care until death or recovery. Neurological outcomes were assessed using the CPC at hospital discharge and at one and six months after OHCA. Follow-up assessments were conducted through in-person visits or standardized telephone interviews with the patient or their primary caregiver.

### 2.3. Data Collection

Clinical data available during the immediate post-cardiac arrest period were extracted from the KORHN-PRO registry. The collected variables included demographic information (age, sex), medical history, cause of arrest, witness status, provision of bystander cardiopulmonary resuscitation (CPR), and initial documented cardiac rhythm. Additional data included resuscitation duration and laboratory findings from initial arterial blood gas analysis, such as pH and lactate levels. Neurological assessments during the immediate post-cardiac arrest period were also recorded, including the presence of ocular reflexes (pupillary and corneal reflexes) and GCS scores. The primary endpoint was a poor neurologic outcome at one month after cardiac arrest, defined as a CPC score of 3–5.

### 2.4. Statistical Analysis

Continuous variables were presented as medians with interquartile ranges (IQRs), due to their non-normal distribution assessed by the Kolmogorov–Smirnov test. Categorical variables were summarized as absolute numbers and percentages. Comparisons of demographic and clinical characteristics between patients with good and poor neurologic outcomes at one month after OHCA were performed using the Mann–Whitney U test for continuous variables and chi-square or Fisher’s exact tests for categorical variables, as appropriate.

To assess the independent prognostic value of additional clinical variables beyond those included in the original rCAST score, multivariable logistic regression analysis was conducted with the rCAST total score incorporated into the model as an offset term. This analytical strategy enabled evaluation of the unique contribution of candidate predictors while adjusting for the baseline rCAST score. Variables considered as potential confounders and tested in univariable analysis included age, sex, and arterial blood gas parameters, such as partial pressure of arterial oxygen (PaO_2_), partial pressure of arterial carbon dioxide (PaCO_2_), and bicarbonate (HCO_3_^−^), and brainstem reflexes, specifically pupillary and corneal reflexes. Other clinical characteristics, such as medical history of hypertension, diabetes mellitus, myocardial infarction, and chronic heart failure, were not included in the analysis, as they are not considered readily available or objective parameters in the immediate post-resuscitation period. Although the cause of arrest is an important prognostic factor, it was also excluded due to the limited reliability of initial etiologic assessment during the immediate post-resuscitation period. Furthermore, as the initial arrest rhythm is a major determinant of presumed cardiac etiology and is already incorporated into the original rCAST score, we considered this factor sufficiently accounted for. The results were reported as odds ratios (ORs) with corresponding 95% confidence intervals (CIs).

Although arterial blood gas parameters, including PaO_2_, PaCO_2_, and bicarbonate, demonstrated significant associations with neurological outcomes in univariable analyses, they were excluded from the final multivariable model due to inconsistent effect directions and evidence of multicollinearity. This exclusion was undertaken to enhance model robustness and clinical interpretability. Based on the final multivariable model, the mCAST score was derived by incorporating the identified independent predictors into the original rCAST framework. Regression coefficients from the final model were used to assign weights to these additional variables in constructing the mCAST score.

The discriminative performance of both the rCAST and mCAST scores was evaluated using receiver operating characteristic (ROC) curve analysis, and areas under the curve (AUCs) were compared using the DeLong test. In reference to a previous study that classified patients into three severity grades (low, moderate, and high) based on the rCAST score, we defined corresponding cutoff points in the mCAST score to achieve 95% sensitivity and 95% specificity [11]. All reported *p*-values were two-sided, and *p*-value of <0.05 was considered statistically significant. All statistical analyses were performed using SAS version 9.4 (SAS Institute Inc., Cary, NC, USA), IBM SPSS Statistics for Windows, version 21.0 (IBM Corp., Armonk, NY, USA), and MedCalc^®^ Statistical Software version 20.118 (MedCalc Software Ltd., Ostend, Belgium; https://www.medcalc.org (accessed on 2 September 2024); 2022).

## 3. Results

**Figure 1:**
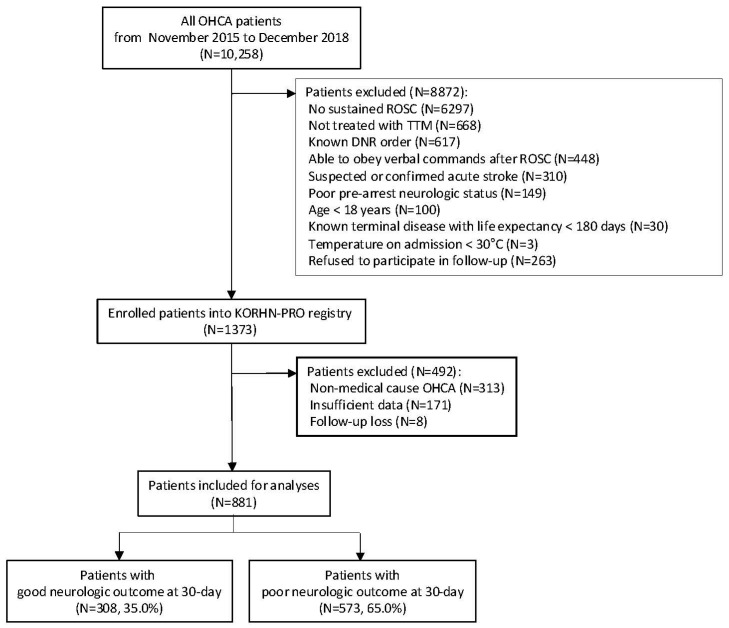
Flow diagram of the patient selection process. Abbreviations: DNR, do not resuscitate; OHCA, out-of-hospital cardiac arrest; ROSC, return of spontaneous circulation.

From among the 1373 comatose OHCA survivors enrolled in the KORHN-PRO registry, 881 patients were included in the final analysis in this present study after excluding cases with a non-medical etiology of cardiac arrest (*n* = 313), insufficient data to calculate the rCAST score (*n* = 171), and loss to follow-up at one month (*n* = 8) (Figure 1).

The demographic and clinical characteristics of the study population are summarized in Table 1. The median age was 60 years, and 73.2% of the patients were male. Compared to patients with poor neurological outcome, those with a good neurological outcome were significantly younger (median, 55.0 vs. 64.0 years; *p* < 0.001) and had a higher rate of witnessed cardiac arrest (86.6% vs. 71.8%, *p* < 0.001). Initial shockable rhythm was also more common in patients with a good outcome (74.4% vs. 24.8%; *p* < 0.001), and the total collapse time was significantly shorter in these patients (median, 20.0 vs. 35.0 min, *p* < 0.001). Arterial blood gas parameters measured after ROSC also differed significantly between the groups. Patients with good neurological outcomes had a higher median pH (7.22 vs. 7.04, *p* < 0.001) and bicarbonate levels (15.8 vs. 14.8 mmol/L, *p* = 0.01), and lower PaCO_2_ (39.1 vs. 55.0 mmHg, *p* < 0.001) and lactate levels (7.5 vs. 10.6 mmol/L, *p* < 0.001). Neurologic examinations during the immediate post-resuscitation period revealed that absent motor response (GCS motor = 1) and absence of pupillary and corneal reflexes were observed at approximately twice the rate in the poor neurological outcome group (87.3% vs. 49.7% and 79.2% vs. 37.7%, respectively; both *p* < 0.001).

To identify additional prognostic factors beyond the rCAST score, we performed univariable logistic regression analyses with the rCAST score included as an offset term (Table 2). An older age was significantly associated with poor neurological outcomes, both as a continuous variable (OR, 1.113; 95% CI, 1.090–1.136; *p* < 0.001) and when dichotomized at >65 years (OR, 12.288; 95% CI, 6.660–22.674; *p* < 0.001). Among the arterial blood gas parameters, lower PaO_2_ (OR, 0.962; 95% CI, 0.951–0.972; *p* < 0.001), higher PaCO_2_ (OR, 1.005; 95% CI, 1.003–1.007; *p* < 0.001), and elevated bicarbonate levels (OR, 1.272; 95% CI, 1.209–1.338; *p* < 0.001) were all significantly associated with poor neurological outcome. The absence of pupillary and corneal reflexes during the immediate post-resuscitation period was also found to be a significant predictor (OR, 1.842; 95% CI, 1.059–3.204; *p* = 0.03).

**Figure 2:**
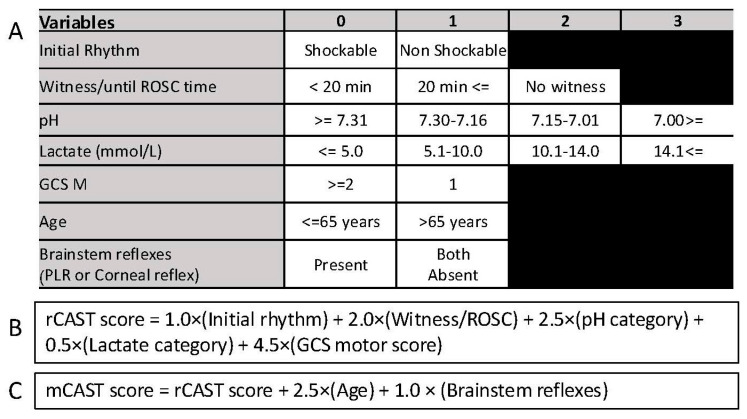
Summary of the rCAST score and the mCAST score. (**A**) Categorization of the variables used in rCAST and mCAST. Variables in the upper five rows (Initial rhythm, Witness/ROSC time, pH, Lactate, GCS motor score) are components of the original rCAST score, while age and brainstem reflexes (bottom two rows) were evaluated separately and added in the mCAST model. (**B**) Calculation formula for the rCAST score. (**C**) Calculation formula for the mCAST score. Abbreviations: GCS M, motor scale of Glasgow Coma Scale; mCAST, modified version of the revised post–Cardiac Arrest Syndrome for Therapeutic hypothermia; rCAST, revised post–Cardiac Arrest Syndrome for Therapeutic hypothermia; PLR, pupillary light reflex.

**Figure 3:**
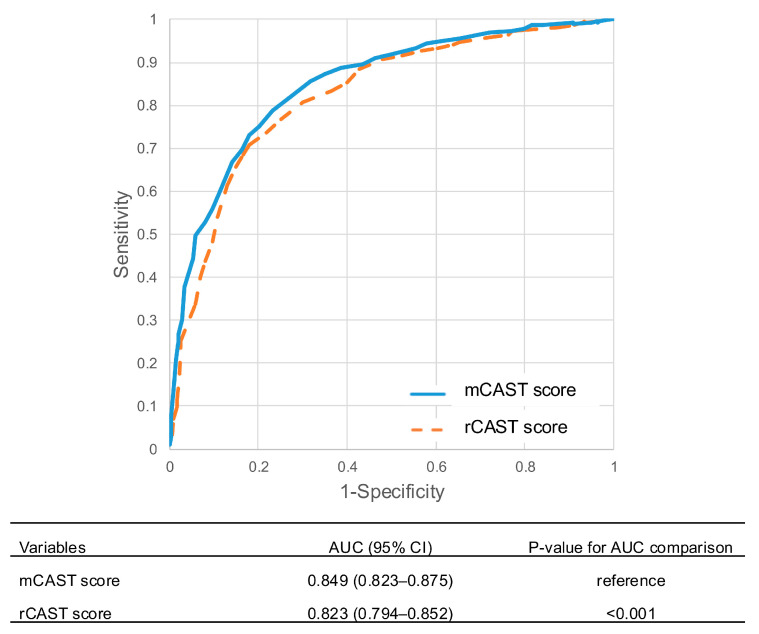
Receiver operating characteristic (ROC) curves for the prediction of poor neurological outcomes at one month using the rCAST score and the mCAST score. Abbreviations: AUC, area under the curve; CI, confidence interval; mCAST, modified version of the revised post–Cardiac Arrest Syndrome for Therapeutic hypothermia; rCAST, revised post–Cardiac Arrest Syndrome for Therapeutic hypothermia.

In the final multivariable logistic regression model incorporating the rCAST score as an offset term, an age > 65 years (OR 13.869, 95% CI 7.377–26.075; *p* < 0.001) and absence of pupillary and corneal reflexes (OR 2.305, 95% CI 1.290–4.118; *p* = 0.005) were identified as independent predictors of a poor neurological outcome (Table 3). Based on the regression coefficients, these variables were incorporated into the mCAST score by assigning 2.5 points for age > 65 and 1.0 point for the absence of brainstem reflexes (Figure 2). The total mCAST score ranges from 0 to 22, compared to 0 to 18.5 for the original rCAST score. Significantly, the mCAST score (AUC, 0.849; 95% CI, 0.823–0.875) demonstrated superior discriminative ability for predicting poor neurological outcomes at one month compared to the rCAST score (AUC, 0.823; 95% CI, 0.794–0.852; *p* < 0.001) (Figure 3).

Table 4 presents the predicted and observed rates of poor neurological outcomes at one month according to the severity classifications derived from the rCAST and mCAST scores. Using the rCAST score, patients were stratified into three risk categories: low severity (score ≤ 5.5; *n* = 145, 16.5%), moderate severity (score 6.0–14.0; *n* = 460, 52.2%), and high severity (score ≥ 14.5; *n* = 276, 31.3%). The corresponding observed rates of poor neurological outcome in each group were 23.4%, 62.6%, and 90.9%, respectively. In contrast, the mCAST score classified patients into low (score ≤ 7.0; *n* = 162, 18.4%), moderate (score 7.5–17.0; *n* = 537, 61.0%), and high (score ≥ 17.5; *n* = 182, 20.7%) severity groups. The observed rates of poor neurological outcome in these groups were 19.8%, 68.5%, and 95.1%, respectively.

## 4. Discussion

In this multicenter observational study of comatose OHCA survivors treated with TTM, we developed the mCAST score, a modified version of the rCAST score, by incorporating an age > 65 years and absence of brainstem reflexes during the immediate post-resuscitation period as additional predictors. The mCAST score demonstrated superior discriminatory performance for predicting poor neurological outcomes at one month compared to the original rCAST score. Moreover, the mCAST allowed for more precise risk stratification by identifying a higher proportion of poor outcomes within a smaller subset of patients classified as high severity. These findings suggest that the mCAST score may enhance early prognostic assessment and support clinical decision-making by more accurately distinguishing patients across prognostic strata.

The performance of the rCAST score in our cohort (AUC = 0.823) was slightly lower than the AUC of 0.892 originally reported in the prior development study of Nishikimi et al. [11]. However, our findings are consistent with results from an external validation study in a U.S. OHCA cohort (AUC = 0.815) and a recent meta-analysis (AUC = 0.84), thereby supporting the generalizability and validity of our dataset [11,12]. While the rCAST score has been previously recognized for its simplicity and practical utility in early prognostication following OHCA [3], there remains a need to improve its predictive accuracy without compromising feasibility. The present study addressed this need by refining the rCAST model through the integration of two clinically meaningful variables. Although the absolute improvement in AUC (ΔAUC = 0.026) may appear modest, this enhancement translated into a more precise reclassification of severity groups and significantly improved identification of high-risk patients, which is critical in early clinical decision-making.

Age and brainstem reflexes were selected based on both clinical plausibility and statistical significance in multivariable analysis. Numerous prognostic tools, such as the rCAST, OHCA, cardiac arrest hospital prognosis (CAHP), and TTM scores, have been developed to stratify the severity of ischemic brain injury in OHCA survivors and to identify patients most likely to benefit from post-resuscitation care [11,13,14,15]. These models typically share core predictors such as initial rhythm, no-flow and low-flow time, and other laboratory variables due to their early availability and prognostic strength [16]. Age is among the most consistently validated predictors of neurological prognosis and is incorporated into both the CAHP and TTM scores, although it was notably absent from the original rCAST. Brainstem reflexes, including pupillary and corneal responses, serve as early indicators of brainstem function and correlate strongly with neurological outcomes [17]. They have also been incorporated into prognostic models such as the TTM and MIRACLE2 scores [15,18]. Their inclusion in mCAST score strengthens its neurobiological relevance and does so without undermining its clinical practicality, as both variables are rapidly and reliably assessable in the early post-resuscitation phase. While classical predictors such as time from collapse to witness or comorbidity profiles have established prognostic value, they were not included in our model. Our primary aim was to develop a simple and practical tool for use in the emergency department. To achieve this, we focused on variables that are immediately available and objectively measurable during the early post-resuscitation period.

The mCAST score achieved improved reclassification across severity categories compared with the rCAST score. Specifically, the proportion of patients classified as high severity was reduced (20.7% vs. 31.3%), while predictive accuracy within this group increased (observed poor outcome rate: 95.1% vs. 90.9%). Additionally, a greater proportion of patients were assigned to the moderate-severity category (61.0% vs. 52.2%), potentially reflecting better resolution in the intermediate risk stratum. The mCAST also slightly improved the identification of patients with favorable outcomes in the low-severity group (good outcome rate: 80.2% vs. 76.6%). These refinements may help mitigate the risk of premature pessimistic prognostication and reduce the inappropriate withholding of aggressive care in patients with potential for recovery. Importantly, these improvements were achieved without sacrificing the simplicity and clinical feasibility that characterized the original rCAST score, thereby enhancing its utility in guiding individualized post-arrest care and optimizing resource allocation.

A notable strength of this present study was that outcome-related bias was likely minimized, as the WLST was legally prohibited in South Korea until February 2018. This legal restriction helped reduce the risk of self-fulfilling prophecy bias, thereby enhancing the validity of the observed neurological outcomes and improving the reliability of prognostic performance assessments. However, several limitations of our current analyses should be acknowledged. First, although we employed a large multicenter cohort, the retrospective and observational design of the study may have introduced unmeasured confounders. Second, the mCAST model was not externally validated in independent cohorts, particularly in cases involving different ethnicities and treatment contexts. Future prospective studies across diverse populations are warranted to confirm its generalizability. Third, although several arterial blood gas parameters (PaO_2_, PaCO_2_, bicarbonate) were associated with outcomes in univariable analyses, they were excluded from the final model due to collinearity with existing rCAST components such as pH and lactate, to improve model parsimony and interpretability. Fourth, inter-institutional variability in post-resuscitation care practices may have influenced outcomes despite overall protocol standardization for TTM. Lastly, although our dataset predates the TTM2 trial and the 2024 International Liaison Committee on Resuscitation recommendations, we emphasize that early clinical markers such as age and brainstem reflexes retain their prognostic relevance independent of specific TTM protocols. Our goal is to inform early-phase risk assessment in the emergency department, rather than replace the comprehensive prognostic strategies applied at 72 h or beyond.

## 5. Conclusions

The mCAST score, developed by integrating age and brainstem reflexes into the original rCAST model, demonstrated enhanced prognostic accuracy for predicting poor neurological outcomes among comatose OHCA survivors. In addition to its improved discriminatory performance, the mCAST score may offer more precise risk stratification by identifying a higher proportion of poor outcomes within a smaller high-severity subgroup. These enhancements were achieved without compromising clinical feasibility, thereby supporting the mCAST score’s potential utility in early post-resuscitation decision-making. External validation in diverse populations and prospective evaluation of its clinical utility are warranted to confirm its generalizability and real-world impact. Continued refinement of early prognostic models such as the mCAST remains essential to guide individualized care and to optimize the allocation of critical care resources following cardiac arrest.

## Figures and Tables

**Table 1:** Demographic and clinical characteristics of the study patients that had undergone an out-of-hospital cardiac arrest.

Characteristic	Total (*n* = 881)	Patients with Good Neurological Outcomes at 1 Month (*n* = 308)	Patients with Poor Neurological Outcomes at 1 Month (*n* = 573)	*p*-Value
Age, years	60.0 (50.0–71.0)	55.0 (44.0–63.0)	64.0 (55.0–74.0)	<0.001
Age > 65 years	327 (37.1%)	63 (20.5%)	264 (46.1%)	<0.001
Male	645 (73.2%)	244 (79.2%)	401 (70.0%)	0.003
Medical history				
Hypertension	356 (40.4%)	90 (29.2%)	266 (46.4%)	<0.001
Diabetes Mellitus	230 (26.1%)	47 (15.3%)	183 (31.9%)	<0.001
Myocardial infarction	62 (7.0%)	20 (6.5%)	42 (7.3%)	0.644
Chronic heart failure	39 (4.4%)	10 (3.2%)	29 (5.1%)	0.212
Chronic kidney disease	72 (8.2%)	11 (3.6%)	61 (10.6%)	<0.001
Witnessed arrest	676 (77.0%)	266 (86.6%)	410 (71.8%)	<0.001
Bystander cardiopulmonary resuscitation	549 (62.3%)	215 (69.8%)	334 (58.3%)	0.001
Initial shockable rhythm	371 (42.1%)	229 (74.4%)	142 (24.8%)	<0.001
Cardiac etiology	704 (79.9%)	294 (95.5%)	410 (71.6%)	<0.001
Time to ROSC, min	28.0 (17.0–43.0)	20.0 (13.0–27.0)	35.0 (22.0–48.0)	<0.001
Arterial blood gas parameters after ROSC				
pH	7.10 (6.94–7.24)	7.22 (7.09–7.30)	7.04 (6.89–7.18)	<0.001
paO_2_ (mmHg)	107.0 (72.3–192.4)	107.0 (73.0–185.8)	107.3 (71.0–195.2)	0.811
paCO_2_ (mmHg)	47.0 (36.0–68.0)	39.1 (32.3–48.9)	55.0 (39.0–75.9)	<0.001
Lactic acid (mmol/L)	9.6 (6.0–12.9)	7.5 (4.7–11.2)	10.6 (7.2–13.6)	<0.001
HCO_3_ (mmol/L)	15.2 (11.9–18.9)	15.8 (12.8–19.4)	14.8 (11.5–18.7)	0.010
GCS motor, 1	653 (74.1%)	153 (49.7%)	500 (87.3%)	<0.001
Absent pupillary and corneal reflexes	570 (64.7%)	116(37.7%)	454 (79.2%)	<0.001
rCAST score	12.0 (8.0–15.0)	7.5 (4.5–10.4)	13.5 (4.5–10.4)	<0.001
Good neurological outcome at 6 months	309 (35.1%)	304 (98.7%)	5 (0.9%)	<0.001

Values are expressed as median (interquartile ranges), mean (standard deviation), or percentage values as appropriate. Abbreviations: GCS, Glasgow coma scale; HCO_3_, bicarbonate; PaO_2_, partial pressure of arterial oxygen; PaCO_2_, partial pressure of arterial carbon dioxide; ROSC, return of spontaneous circulation.

**Table 2:** Univariable logistic regression analysis for individual predictors of poor neurological outcome with the rCAST score included as an offset term.

Characteristic	Odds Ratio	95% Confidence Interval	*p*-Value
Age, years	1.113	1.090–1.136	<0.001
Age > 65 years	12.288	6.660–22.674	<0.001
Male	0.612	0.440–0.490	0.003
Initial arterial blood gas parameters			
PaO_2_	0.962	0.951–0.972	<0.001
PaCO_2_	1.005	1.003–1.007	<0.001
HCO_3_	1.272	1.209–1.338	<0.001
Absent pupillary and corneal reflexes	1.842	1.059–3.204	0.03

Abbreviations: HCO_3_, bicarbonate; PaO_2_, partial pressure of arterial oxygen; PaCO_2_, partial pressure of arterial carbon dioxide; rCAST, revised Cardiac Arrest Syndrome for Therapeutic hypothermia.

**Table 3:** Final multivariable logistic regression model for poor neurological outcome with the rCAST score included as an offset term.

Characteristic	Adjusted Odds Ratio	95% Confidence Interval	*p*-Value	Estimate (Beta)	Standard Error
Age > 65 years	13.869	7.377–26.075	<0.001	1.3148	0.161
Absent pupillary and corneal reflexes	2.305	1.290–4.118	0.005	0.4176	0.148
rCAST score				1	0

Abbreviations: rCAST, revised Cardiac Arrest Syndrome for Therapeutic hypothermia.

**Table 4:** Predicted probability of poor neurological outcome and actual patient outcomes at 1 month according to rCAST and mCAST severity classifications.

Severity Classifications	Number	Probability of Poor Neurological Outcome (95% CI)	Actual Poor Neurologic Outcome, *n* (%)
rCAST			
Low (≤5.5)	145 (16.5%)	23.5 (17.3–31.0)	34 (23.4%)
Moderate (6–14)	460 (52.2%)	62.6 (58.1–66.9)	288 (62.6%)
High (≥14.5)	276 (31.3%)	90.9 (86.9–93.8)	251 (90.9%)
mCAST			
Low (≤7.0)	162 (18.4%)	30.3 (25.5–35.9)	32 (19.8%)
Moderate (7.5–17.0)	537 (61.0%)	67.3 (63.7–70.8)	368 (68.5%)
High (≥17.5)	182 (20.7%)	89.1 (86.0–91.6)	173 (95.1%)

Abbreviations: CI, Confidence interval; mCAST, modified Cardiac Arrest Syndrome for Therapeutic hypothermia; rCAST, revised Cardiac Arrest Syndrome for Therapeutic hypothermia.

## Data Availability

The datasets used and/or analyzed during the current study are available from the corresponding author on reasonable request.

## Abbreviations

The following abbreviations are used in this manuscript:

AUC	Area Under the Curve
CAHP	Cardiac Arrest Hospital Prognosis
CI	Confidence interval
CPC	Cerebral Performance Category
CPR	Cardiopulmonary resuscitation
GCS	Glasgow Coma Scale
KORHN-PRO	Korean Hypothermia Network prospective registry
mCAST	modified version of the revised post–Cardiac Arrest Syndrome for Therapeutic hypothermia
OHCA	Out-of-hospital cardiac arrest
OR	Odds ratio
PaO_2_	Partial pressure of arterial oxygen
PaCO_2_	Partial pressure of arterial carbon dioxide
rCAST	revised post–Cardiac Arrest Syndrome for Therapeutic hypothermia
ROC	Receiver operating characteristic
ROSC	Return of spontaneous circulation
TTM	Targeted temperature management
WLST	Withdrawal of life-sustaining treatment
